# Chromatin Remodeling in VSMC Phenotype Switching During Vascular Remodeling: From Mechanism to Therapeutic Potential

**DOI:** 10.3390/biom16020265

**Published:** 2026-02-07

**Authors:** Xiaozhu Ma, Shuai Mei, Qidamugai Wuyun, Li Zhou, Hu Ding, Jiangtao Yan

**Affiliations:** 1 Division of Cardiology, Department of Internal Medicine, Tongji Hospital, Tongji Medical College, Huazhong University of Science and Technology, 1095# Jiefang Ave, Wuhan 430030, China; xzma2023@hust.edu.cn (X.M.); 2025tj0120@hust.edu.cn (S.M.); d202282253@hust.edu.cn (Q.W.); d202582786@hust.edu.cn (L.Z.); 2 Hubei Key Laboratory of Genetics and Molecular Mechanisms of Cardiological Disorders, Wuhan 430030, China; 3 Key Laboratory of Vascular Aging, Ministry of Education, Tongji Hospital of Tongji Medical College, Huazhong University of Science and Technology, Wuhan 430030, China

**Keywords:** chromatin remodeling, VSMC phenotypic switching, histone modification, vascular remodeling

## Abstract

Vascular remodeling is a characteristic pathological feature of various vascular diseases, including atherosclerosis, restenosis following vascular injury, hypertension, and aneurysms. The phenotypic switching of vascular smooth muscle cells (VSMCs) acts as a key driver of vascular remodeling. Under specific pathological stimuli, VSMCs rapidly transition from a contractile to a dedifferentiated phenotype, characterized by enhanced proliferation, migration, and secretory activity. Chromatin remodeling, a core mechanism of epigenetic regulation, orchestrates dynamic changes in chromatin structure and function through ATP-dependent remodeling complexes, histone-modifying enzymes, and DNA methyltransferases. These components collectively translate mechanical stress, metabolic disturbances, and inflammatory signals into reversible epigenetic modifications, thereby precisely regulating VSMC phenotypic switching. As such, chromatin remodeling represents a critical node for therapeutic intervention in vascular remodeling-related diseases. In recent years, a growing body of research has focused on the role of chromatin remodelers in regulating VSMC phenotype. In this review, we focus on the roles of ATP-dependent chromatin-remodeling factors and chromatin-modifying enzymes in the control of gene expression of VSMC phenotype switching. Firstly, we summarize the latest insights into chromatin remodeling and VSMC phenotypic switching, and then discuss recent advances in the identification and functional characterization of chromatin remodeling molecules, emphasizing their implications for VSMC behavior. Finally, we highlight the translational potential of targeting chromatin remodelers in the development of clinical therapies for vascular remodeling diseases and outline future directions for research in this field.

## 1. Introduction

Pathological vascular remodeling, which is defined by intimal hyperplasia, medial thickening and arterial stiffness, represents a primary pathological manifestation of cardiovascular diseases, such as atherosclerosis, hypertension, restenosis following vascular injury, aneurysms, etc. [1]. Despite widespread deployment of lipid-lowering and antihypertensive therapies, cardiovascular disease continues to impose the greatest global burden of illness and mortality. The incidence of cardiovascular disease exhibits a persistent upward trajectory, with epidemiological forecasts estimating approximately 35.6 million attributable fatalities by 2050, posing formidable challenges to the management of vascular-related disorders [2,3].

The hallmark of maladaptive vascular remodeling is the remarkable phenotypic plasticity of vascular smooth muscle cells (VSMCs). Unlike terminally differentiated skeletal or cardiac muscle, mature VSMCs retain a reversible contractile-to-synthetic switch that can be re-engaged within hours after endothelial denudation, sustained hypertension, or metabolic stress [4]. This transition is characterized by down-regulation of contractile genes encoding α-smooth muscle actin (ACTA2), transgelin (TAGLN/SM22α), calponin-1 (CNN1), myosin heavy chain 11 (MYH11), and the master co-activator myocardin (MYOCD), and by concomitant up-regulation of synthetic, migratory, and secretory programs mediated by Krüppel-like factor 4 (KLF4), osteopontin (OPN), and matrix metalloproteinases (MMPs) [5]. Recently, single-nucleus RNA-seq reveals that this transition is not unidirectional terminal differentiation but rather a highly heterogeneous and reversible dynamic spectrum, enabling VSMCs to oscillate between quiescent, proliferative, inflammatory, and osteochondrogenic states [6]. Consequently, the balance between contractile integrity and adaptive plasticity determines whether vascular repair resolves cleanly or progresses to pathological remodeling.

Traditional research has predominantly concentrated on growth factor signaling and transcription factor regulation, yet it has encountered challenges in elucidating the establishment of phenotypic memory and the dynamics of rapid switching. Emerging evidence now positions chromatin remodelers as the central metabolic and mechanotransduction hubs that integrate mechanical, oxidative, and inflammatory signals to direct long-term VSMC fate [7,8,9]. The association between mechanical cues and chromatin remodeling events in VSMCs is dependent on an integrated structural network spanning from the extracellular space to the nuclear interior. Stress fibers in contractile VSMCs attach to the nuclear envelope via molecular bridges, notably the LINC (Linker of Nucleoskeleton and Cytoskeleton) complex [10]. This connection enables force transmission from the extracellular matrix to the nuclear interior, where it induces structural and functional changes in chromatin organization [11,12]. Mannion, A. J. et al. found that uncoupling of the nucleus from the cytoskeleton induces aberrant nuclear morphology, reduced chromatin accessibility, and YAP (Yes-associated protein) promoter activity in blood vessels [13]. In addition, the nuclear lamina undergoes structural reorganization, which is characterized by the localized enrichment of A-type lamins and decondensation of chromatin domains at force-transmitting sites, thereby modulating the accessibility of specific genomic loci [14]. Therefore, systematic investigation of dynamic recruitment of chromatin remodelers, post-translational modification, and small-molecule druggability is poised to illuminate the previously enigmatic establishment of phenotypic memory and rapid switching capacity of VSMCs.

Accordingly, in this review, we briefly elucidate the fundamental principles underlying chromatin remodeling and VSMC phenotypic transition. Then we discuss the most significant advances in chromatin remodeling molecules involved in VSMC phenotypic switching and examine the translational potential of chromatin-remodeling factors, aiming to provide a theoretical foundation for the treatment of diseases associated with vascular remodeling.

## 2. Overview of Chromatin Remodeling

Nucleosomes, the fundamental repeating units of chromatin, are composed of 146 base pairs of DNA wrapped around an octamer of core histones [15]. This configuration compacts genomic DNA while simultaneously imposing both physical and electrostatic barriers that restrict access to underlying regulatory elements. Consequently, gene transcription necessitates localized chromatin decondensation, enhanced DNA accessibility, and the coordinated recruitment of sequence-specific transcription factors and the RNA polymerase II machinery [16]. To reconcile genome packaging with transcriptional plasticity, eukaryotic organisms have evolved sophisticated regulatory networks centered on post-translational modifications (PTMs) of histone N-terminal tails [17]. These evolutionarily conserved residues serve as substrates for diverse chromatin-modifying enzymes, giving rise to an array of PTMs, including acetylation, methylation, phosphorylation, lactylation, and ubiquitination, collectively constituting an epigenetic code [18]. This combinatorial modification landscape modulates higher-order chromatin architecture and plays pivotal roles in DNA replication, chromatin assembly, and the precise temporal and spatial regulation of gene expression.

Chromatin remodeling, defined as the ATP-dependent repositioning, ejection, or exchange of nucleosomes, represents a fundamental epigenetic mechanism that regulates DNA accessibility without changing the primary sequence [19]. This process encompasses modifications in DNA methylation, histone marks, and the activity of ATP-dependent chromatin remodelers. The basics of DNA methylation and histone modification have been summarized in our previous review [20]. Chromatin-remodeling factors catalyze nucleosome assembly, sliding, eviction, or histone variant exchange by harnessing the energy released from ATP hydrolysis, thereby reprogramming local chromatin architecture to govern transcriptional output and orchestrate developmental and metabolic processes [21]. In mammals, there are four major families about these ATP-depended chromatin remodeling factors: Switch defective/sucrose non-fermentable (SWI/SNF), imitations switch (ISWI), chromodomain helicase DNA binding protein (CHD), and INOsitol requiring 80 (INO80) [22]. They share a conserved ATP domain motor that combines with multiple non-ATPase subunits to form chromatin remodeling complexes. Divergent auxiliary domains confer distinct biochemical outputs, enabling specific control of chromatin topology and homeostasis. The SWI/SNF family functions as a principal ATP-driven nucleosome disruptor: hydrolysis-dependent DNA translocation loosens histone-DNA contacts, slides nucleosomes and generates nucleosome-free regions that license transcription factor binding and RNA-polymerase-II initiation [23]. These activities position SWI/SNF as the remodeler most intimately connected with gene expression control [24]. The core subunit of SWI/SNF is the ATP-dependent catalytic subunit SMARCA4/2 (also known as brahma-related gene 1 (BRG1) and brahma (BRM)), and mutations in different subunit genes are closely related to development and tumorigenesis [25,26]. ISWI enzymes sense linker length and histone marks to space nucleosomes regularly, folding chromatin and establishing heterochromatin boundaries during replication and mitosis [27,28,29]. The CHD family exhibits biological functions akin to those of ISWI in regulating nucleosome gliding and chromatin status [30]. However, it places greater emphasis on mediating gene activation or inhibition through the recognition of histone modification states and involvement in chromatin assembly and remodeling. INO80 not only senses nucleosome spacing and regulates nucleosome gliding but also coordinates with SWI/SNF to modulate chromatin accessibility. Additionally, it catalyzes the exchange of histone dimers H2A-H2B and H2A [31]. The exchange of Z-H2B variant dimers promotes the dynamic insertion and removal of H2A.Z.

**Figure 1:**
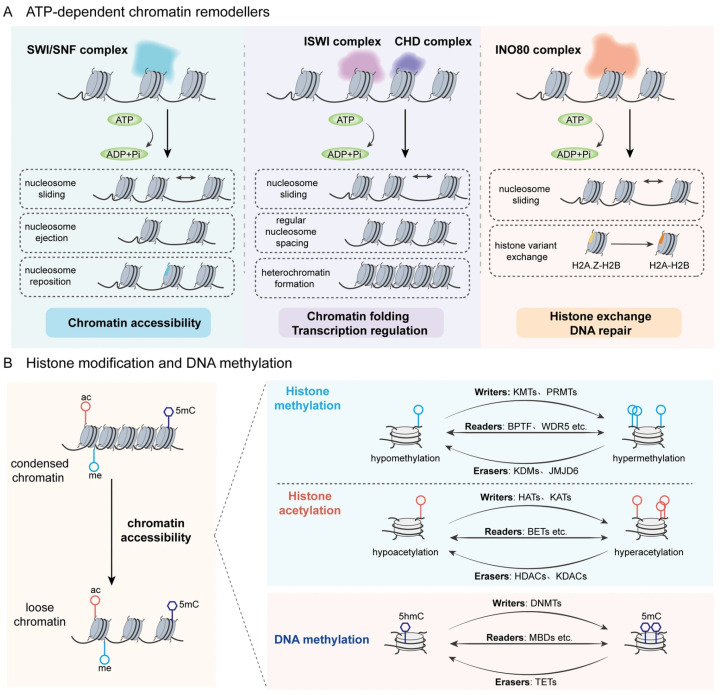
Classification and mechanisms of major chromatin remodelers. (**A**) Chromatin remodeling is fundamentally dependent on ATP-dependent chromatin-remodeling complexes, histone-modifying enzymes, and DNA methylation. Mammalian ATP-dependent chromatin-remodeling complexes are classified into four major groups: SWI/SNF, ISWI, CHD, and INO80. By harnessing the energy released from ATP hydrolysis, they induce structural rearrangements in chromatin. SWI/SNF maintains regulatory regions in an open state by sliding, ejecting, or reorganizing nucleosomes. ISWI and CHD are structurally similar. ISWI senses linker DNA length to establish uniform nucleosome spacing and is essential for heterochromatin formation and genomic stability, whereas CHD recognizes diverse histone modifications, thereby recruiting or evicting specific proteins to modulate transcription. INO80 binds and translocates hexasomes to catalyze histone variant exchange, a process intimately linked to DNA replication, transcription, and repair. (**B**) Chromatin accessibility is also inseparable from covalent histone modifications and DNA methylation. Among the myriad of histone marks, methylation and acetylation are the most extensively characterized. The balanced state of these modifications is dynamically maintained by the concerted action of writers (that promote modification formation), readers (that recognize modification sites), and erasers (that remove or inhibit modification formation). Some representative enzymes are illustrated in the figure.

It is significant to note that chromatin-remodeling complexes do not function independently. Their regulation of chromatin homeostasis is dependent on dynamic interactions with DNA methylation and histone modifications. Remodelers reconfigure local chromatin architecture, thereby increasing the accessibility of DNA to methyltransferases and histone tails to modify enzymes. Conversely, covalent histone marks not only modulate nucleosomal stability by altering histone-histone and histone-DNA contacts, but also serve as docking platforms that recruit specific remodeling complexes, establishing a self-reinforcing regulatory circuit [32]. For instance, SWI/SNF complexes physically interact with CBP/p300 to catalyze histone H3 lysine 27 acetylation (H3K27ac), potentiating enhancer activation [33]. The resultant H3K27ac is recognized by the bromodomain of BRG1 (SMARCA4), which in turn stabilizes mSWI/SNF occupancy at target loci to further amplify H3K27ac, constituting a positive-feedback loop. The nucleosome remodeling and deacetylase (NuRD) complex, a canonical chromatin-repressive assembly, plays an important role in development, stem cell biology, and DNA repair [34]. It comprises the remodelers CHD3/4 (Mi-2α/β), the deacetylases HDAC1/2, and specificity modules. CHD3/4 provides ATP-dependent nucleosome sliding or ejection, whereas HDAC1/2 removes acetyl moieties from H3/H4 tails, partially restoring positive charge to compact chromatin and reinforcing the heterochromatic state [35,36]. Additionally, the NuRD platform recruits DNA methyltransferase (DNMT3A/B) to direct CpG hypermethylation and consolidate transcriptional silencing [37,38]. Although its high-resolution architecture remains incomplete, the coordinated deacetylation, remodeling and DNA-methylation activities of NuRD constitute definitive evidence for tripartite crosstalk among chromatin-modifying systems (Figure 1).

Notably, the regulation of gene expression encompasses multiple layers. As previously discussed, the local chromatin environment is orchestrated by the synergistic regulation of histone post-translational modifications, DNA methylation, and alterations in nucleosome positioning. These modifications facilitate the recruitment of chromatin-remodeling factors or transcription factors to specific DNA sites, thereby inducing transcriptional activation or repression [39]. Moreover, the pivotal role of three-dimensional chromatin structure in the regulation of gene expression cannot be overlooked. Higher-order chromatin structures exhibit diverse forms, including DNA loops, topologically associating domains (TADs), and chromosome territories [40]. DNA loops, essential structural components of chromatin architecture formed by interactions between distant genomic regions mediated by the cohesin complex, enable spatial proximity between enhancers and promoters, thereby enhancing transcriptional activity [41]. TADs are higher-order chromatin structures that consist of multiple DNA loops. The interactions with TAD occur more frequently than those with external regions [42]. However, some promoters and enhancers can interact across TAD boundaries [43]. Chromosome territories represent the highest level of chromatin organization within the nucleus, with each chromosome occupying a distinct spatial domain that compartmentalizes the genome [44]. Based on their chromatin states and functional characteristics, chromosome territories are typically divided into A and B compartments [45]. The A compartment is characterized by euchromatin, which is gene-rich and transcriptionally active, while the B compartment is enriched in heterochromatin, which is transcriptionally inactive. The spatial arrangement is crucial for maintaining genome integrity and facilitating essential nuclear processes, such as DNA replication and transcription [46].

## 3. Diversity of VSMC Phenotypic Switching During Vascular Remodeling

VSMC phenotypic switching is the cellular hallmark of pathological vascular remodeling. The traditional dichotomy of phenotypic switching is based on cellular function and the expression of specific molecular markers. For instance, the synthetic phenotype is characterized by an increased capacity for synthesizing pro-inflammatory factors and extracellular matrix components, which are difficult to quantify [47]. Moreover, under certain pathological conditions, the expression of specific molecular markers may be downregulated, making them challenging to detect and potentially leading to misinterpretation. Therefore, the traditional classification has its limitations. Recent advancements in single-cell sequencing, lineage tracing, and spatial transcriptomics have enabled a deeper investigation into the alterations of tissue cell subpopulations, gene changes in various cell types, and cellular origins under disease conditions [48,49,50,51]. The development of lineage-tracing techniques has further unveiled the complexities of smooth muscle phenotypic switching. By crossing mice harboring a missing marker (a fluorescent reporter transgene) with those expressing smooth muscle contractile genes (such as TAGLN or MYH11), and inducing recombinase expression with tamoxifen, this approach allows for the tracking of VSMCs and their derivatives [52]. Interestingly, the majority of plaque cells originate from the recruitment and proliferation of local VSMCs [53]. These studies have revealed the presence of multiple cell subtypes within atherosclerotic tissues, all of which are derived from a small number of smooth muscle cells.

Recent single-cell and lineage-tracing studies have also revealed that contractile cells first enter a pluripotent “intermediate-dedifferentiated” state that can be diverted, by defined extracellular cues such as growth factors, cytokines, hypoxia and mechanical stress, into multiple types—synthetic VSMCs, osteoblast-like, macrophage-like, mesenchymal-stem-like, myofibroblast, adipocyte and chondrocyte-like cells [54,55,56]. Synthetic VSMCs, which display fibroblast-like morphology, significantly larger volume than contractile VSMCs, initiate the tissue-repair process and are therefore considered “intermediate cells” that subsequently switch to specific phenotypes under different pathological stimuli [57]. The latest pseudo-time analyses divide the traditional VSMC pool into three sequential stages—differentiated, intermediate-dedifferentiated and dedifferentiated [58]. The intermediate-dedifferentiated compartment contains mesenchymal VSMCs, Stem/Endothelial/Monocyte (SEM)-like VSMCs and Lgals3+ cells. Mesenchymal-like cells can differentiate into adipocytes, osteoblasts and macrophage-like cells in atherosclerosis models, yet still revert to contractile VSMCs upon transforming growth factor-beta (TGF-β) treatment. Time-fitting analyses place SEM clusters at the mid-point of the trajectory and constitute the largest VSMC-derived population within plaques and subsequently bifurcate into macrophage-like or osteoblast-like cells that determine plaque stability [56]. Dual-lineage tracing of human and murine late-stage atherosclerotic plaques further shows that 60–80% of VSMC-derived cells have passed through the Lgals3+ phase, indicating that Lgals3+ cells represent an additional intermediate state [59]. The directional fate of intermediate-state smooth muscle cells is influenced by signals from the cellular microenvironment. Osteoblast-like cells are usually found in vascular tissues stimulated by high phosphate and calcium, and macrophage-like cells are mostly found in high glucose and high lipid stimulation [60,61]. Myofibroblasts are commonly driven by cytokines and physicochemical stimuli [62]. In recent studies, under specific vascular disease conditions, smooth muscle cells can be further classified into several distinct subtypes based on specific marker proteins, and these subtypes are referred to as mod-VSMCs or fibromyocytes in atherosclerosis or aortic aneurysm [63,64]. For instance, in aortic aneurysm tissues, smooth muscle cells account for up to 65% of the cell population and are categorized into three subtypes [65]. Research has shown that the more mature and differentiated subpopulation of Nrip2+ smooth muscle cells (SMCs) is crucial for maintaining mitochondrial metabolic homeostasis in smooth muscle cells and vascular homeostasis [66].

Consequently, multiple smooth-muscle phenotypes coexist within the same vascular lesion, creating a multidirectional, parallel pattern that jointly executes the repair response to various vascular injuries.

## 4. Mechanism of VSMC Phenotypic Switching

### 4.1. Transcription Factors

Serum-response factor (SRF) is the principal transcription factor in maintaining VSMC contractility. Via binding to the cis-regulatory element CArG elements in the promoter region of smooth muscle contractile marker genes, SRF either activates transcription by recruiting the co-activator MYOCD or represses it by associating with the co-repressor myocardin-related transcription factor-A (MRTFA) [67]. Under vascular injury or platelet-derived growth factor-BB (PDGF-BB) stimulation, MRTFA is up-regulated and shifts the balance toward repression [68]. A recent study has shown that the stability of SRF protein is partially influenced by small ubiquitin-like modifier (SUMO) modification, which is catalyzed by SUMO-specific protease 1 (SENP1). Deletion of SENP1 enhances the SUMO1 modification of SRF at the K143 site, leading to increased accumulation of SRF in the nucleus and its interaction with phosphorylated Ets-like kinase 1 (ELK1). As a result, the formation of the SRF-MYOCD complex is diminished, which in turn decreases the expression of smooth muscle-specific genes [69]. Additionally, ELK1 facilitates the activation of the staphylococcal nuclease domain-containing protein 1 (SND1) gene, which competes with MYOCD and contributes to an enhanced smooth muscle synthesis phenotype [70].

KLF4, a phylogenetically conserved zinc-finger nuclear protein, antagonizes SRF-dependent contractile gene transcription by competitive occupancy of CArG boxes to preclude SRF-MYOCD-DNA ternary complex assembly [71]. Additionally, KLF4 binds directly to G/C repressor elements and nucleates histone deacetylase 2 (HDAC2), phospho-ELK1, and other co-repressor complexes to establish a transcriptionally silent chromatin environment [72]. Beyond contractile gene repression, KLF4 orchestrates multiple facets of VSMC phenotypic switching, as comprehensively reviewed recently [63].

Apart from the canonical transcriptional regulators described above, an increasing range of newly identified factors have been implicated in VSMC phenotypic switching. The E-box–binding protein Slug has been implicated in VSMC dedifferentiation. Coll-Bonfill, N. et al. [73] demonstrated elevated Slug expression in extensively remodeled pulmonary arteries from both human patients and murine models. Mechanistically, Slug promotes the expression of genes governing proliferation and migration in VSMCs during TNF-α (tumor necrosis factor-α)-induced phenotypic switching toward a pro-inflammatory and hyperproliferative state. In addition, Slug is markedly up-regulated by PDGF and drives inflammatory reprogramming by activating extracellular signal-regulated kinase 1/2 (ERK1/2), enhancing cholesterol efflux and accelerating VSMC migration [74]. BTB and CNC homology 1 (BACH1) occupies the proximal promoters of contractile genes, where it recruits the histone methyltransferase G9a and the mechanotransducer YAP to establish H3K9me2-repressive chromatin, thereby consolidating mechanically induced synthetic phenotypes [75]. Likewise, activating transcription factor 3 (ATF3), a stress-responsive bZIP family member rapidly induced by inflammation, hypoxia or cyclic stretch, targets the PDGFRβ locus to amplify receptor expression, facilitating VSMC dedifferentiation and proliferation [76,77].

### 4.2. Growth Factors

VSMC phenotype is highly susceptible to stimulation by various cytokines, which influence the phenotypic transformation process of smooth muscle through a cascade reaction. PDGF-BB, a peptide growth factor from the PDGF family, is synthesized by smooth muscle cells, macrophages, and other cell types [78]. PDGF-BB affects the expression of genes associated with VSMC phenotype transition through several mechanisms. PDGF-BB activates multiple signaling pathways upon binding to the PDGFR-β, including the ERK1/2, JAK2/STAT3, and the mTOR/p70S6k signaling pathway [79,80,81]. This activation induces key transcription factors, thereby regulating phenotype transition. Additionally, it modulates the expression of endogenous non-coding RNA, influencing gene transcription and translation processes, which subsequently regulate cell proliferation, migration, and phenotypic transformation [82,83].

TGF-β influences the phenotypic transformation of smooth muscle cells via both the classical SMAD signaling pathway and a Smad-independent pathway. Specifically, TGF-β binds to TGF-β receptor I/II on the surface of VSMCs [84]. This binding induces the phosphorylation of downstream Smad2 and Smad3, which subsequently form a complex with Smad4 and translocate into the nucleus. This process upregulates the expression of contraction-related genes while inhibiting the phenotypic transformation of VSMCs. Furthermore, TGF-β signaling can activate the PI3K-Akt-mTOR pathway and the ERK/JNK/p38 pathway, thereby regulating genes associated with smooth muscle contraction, proliferation, and migration. Additionally, proinflammatory molecules such as TNF-α, IFN-γ, IL-11, and angiotensin II have also been employed to explore the mechanisms underlying smooth muscle phenotype conversion [85,86,87,88].

### 4.3. Signaling Pathways

The Notch signaling pathway is an evolutionarily conserved mechanosensitive axis. Notch1-4 are transmembrane receptors that recognize juxtacrine Jagged ligands on adjacent cells, mediate endothelial smooth muscle communication and thereby govern early vascular morphogenesis as well as maintenance of the quiescent contractile phenotype in adult VSMCs [89]. Notch also interlocks with TGF-β/Smad3 and MAPK/ERK pathways to maintain the dynamic balance between smooth muscle phenotypes. In parallel, the Hippo YAP pathway is triggered by PDGF-BB, Ang II or cyclic stretch.

**Figure 2:**
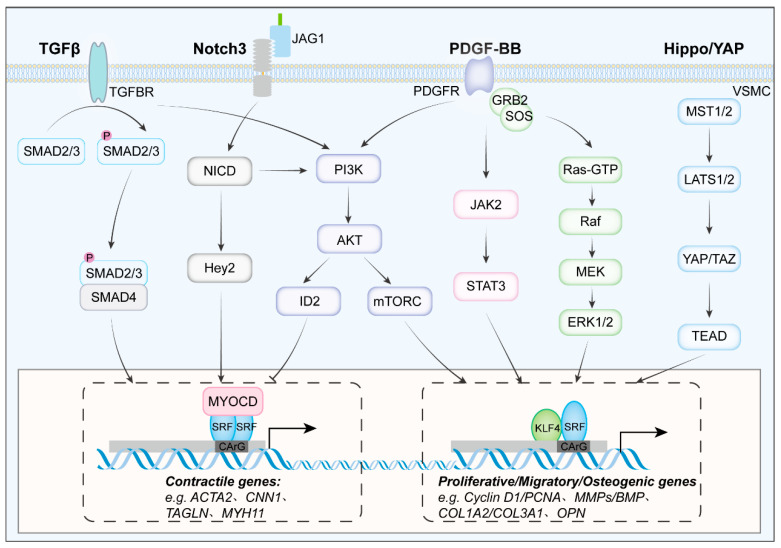
Integrated signaling circuitry governing VSMC phenotypic switching. The TGF-β, PDGF, Notch and Hippo pathways form an interconnected network that dictates the contractile-to-synthetic switch of vascular smooth-muscle cells. TGF-β engages canonical SMAD2/3/4 to sustain MYOCD-SRF-CArG transcriptional activity [92] while concomitantly activating PI3K-AKT-ID2 signal, which promotes VSMCs switch from contractile to synthetic phenotype [93]. JAG1-Notch3 cleavage releases NICD that amplifies Hey2 and curbs excessive synthetic gene expression [94]. PDGF-BB triggers MEK-ERK1/2 and parallel PI3K-Akt-mTORC signaling pathways, de-activating MYOCD and elevating KLF4/STAT3 to repress CArG-box-driven contractile genes [95]. Mechanical or cytokine signals inhibit MST1/2-LATS1/2, facilitating the entry of de-phosphorylated YAP/TAZ into the nucleus, where they associate with TEAD to enhance proliferative, migratory, and osteogenic targets [96].

Activated YAP dislodges the MYOCD-SRF-CArG complex and represses the expression of contractile genes [90]. When the core kinases of the Hippo pathway are silenced, YAP/TAZ become dephosphorylated and translocate into the nucleus, where they interact with TEAD transcription factors. This leads to the transcriptional activation of genes associated with osteogenesis, inflammation, and proliferation, thereby promoting multidirectional VSMC phenotypic switching [91] (Figure 2).

## 5. Chromatin Remodeling in VSMC Phenotype Switching

Given that the majority of existing studies have primarily concentrated on deciphering its pivotal role in the phenotype of smooth muscle cells from the perspective of molecular regulation, in this review, we focus on summarizing the research progress of chromatin remodeling regulatory molecules in the context of VSMC phenotypic switching.

### 5.1. ATP-Dependent Chromatin Remodellers in VSMC Phenotype Switching

The ATP-dependent chromatin remodelers have been investigated in relation to vascular development and multiple vascular remodeling-related diseases, especially the SWI/SNF complexes [97]. The ATPase catalytic subunits of the SWI/SNF complexes, BRG1 and BRM has been the most extensively studied. As early as 2002, a study had indicated that BRG1 was upregulated in atherosclerotic plaques and in models of in-stent restenosis [98]. Over the past two decades, an increasing number of studies have highlighted the role of SWI/SNF in vascular behavior and phenotype switching.

Early investigations revealed that the ATPase subunits BRG1 and BRM could interact with the N-terminus of myocardin and form a complex with myocardin and SRF, thereby facilitating the binding of SRF to gene promoters, which is essential for the expression of smooth muscle marker genes [99]. In addition to directly regulating contractile marker genes, BRG1 can indirectly influence the contractile phenotype by modulating miRNA expression [100]. Subsequently, researchers found that aberrant expression of BRG1 was also implicated in gene expression regulation under disease conditions. Endothelin-1 (ET-1) is a potent vasoconstrictor peptide that promotes cell fibrosis, proliferation, and inflammatory responses [101,102]. BRG1/BRM participates in endothelin-1 (ET-1)-induced contraction and inflammatory responses in smooth muscle cells. Studies have shown that in primary rat smooth muscle cells, BRG1 binds to the promoters of proliferation-related genes, including proliferating cell nuclear antigen (PCNA), neurotrophin 3 (NTF3), and platelet-derived growth factor subunit A (PDGFA), leading to gene activation and participating in ET-1-stimulated cell proliferation [103]. In addition, BRG1 and BRM bind to the promoters of Interleukin 6 (IL6), IL1β, and C-C motif ligand 2 (CCL2), mediating ET-1-stimulated inflammatory responses [104].

Vascular smooth muscle-derived Sca1+ adventitial progenitor (AdvSca1-SM) cells are multipotent cells that can differentiate into myofibroblasts during acute vascular injury, thereby participating in vascular remodeling and repair [105,106]. A recent study found that BRG1 was significantly upregulated in AdvSca1-SM cells after carotid ligation injury. In the early stages of carotid ligation-induced vascular injury, BRG1 promotes the transformation of AdvSca1-SM cells into macrophage-like cells, which participate in the inflammatory response. In the late stages of vascular injury, AdvSca1-SM cells differentiate into myofibroblasts, promoting collagen deposition and vascular remodeling. Treatment with the BRG1 bromodomain small molecule inhibitor PFI-3 significantly ameliorates pathological changes in both the early and late stages of vascular injury [107]. Further Cleavage Under Targets and Release Using Nuclease (CUT&RUN) assays have revealed that the primary mechanism involves decreased binding of BRG1 to the promoters of stemness-related genes and increased binding to myofibroblast-related genes, including ACTA2, POSTN, and COL2A1.

Moreover, BRG1 is upregulated during the osteogenic transformation of smooth muscle cells, both in primary rat aortic smooth muscle cells stimulated by high phosphate and in calcified vessels from dialysis patients [108]. Recent studies have also elucidated the role of BRG1 in vascular aneurysm formation. Li et al. [109] found that BRG1 is upregulated in angiotensin II-induced aneurysmal tissues. BRG1 promotes the transcription of the Cathepsin K (CTSK) gene, leading to increased apoptosis of smooth muscle cells and upregulation of matrix metalloproteinase (MMP) expression. Specific genetic knockout of BRG1 in the vasculature or inhibition of BRG1 activity using small molecules significantly reduces the incidence of vascular aneurysms in mice.

The BRG1/BRM-associated factor 60 (BAF60) family, comprising the core subunits BAF60a, BAF60b, and BAF60c (SMARCD1/2/3), represents a subset of the non-ATPase subunits within the SWI/SNF complex and functions as an essential mediator. These subunits facilitate the recruitment of SWI/SNF complexes to target gene promoters, thereby enabling localized chromatin remodeling and transcriptional regulation [110]. Specifically, BAF60a enhances the association of BRG1, the catalytic subunit of the SWI/SNF complex, with the promoter regions of NF-κB target genes, playing a role in the inflammatory responses of smooth muscle cells [111]. Further investigations have revealed that mice with smooth muscle-specific knockouts of BAF60c exhibit increased susceptibility to angiotensin II or elastase-induced aortic aneurysms, along with heightened elastin degradation, vessel wall inflammation, smooth muscle cell transdifferentiation into macrophages, and apoptosis. Mechanistically, BAF60c sustains the contractile phenotype of smooth muscle cells by facilitating the interaction between SRF and the coactivator p300. Additionally, BAF60c preserves chromatin repression around NF-κB target genes through the mediation of HDAC1, thereby inhibiting the inflammatory transition of smooth muscle cells [112].

INO80, the catalytic core of the evolutionarily conserved INO80 chromatin-remodeling complex, has emerged as a pleiotropic regulator of cell fate that operates at the intersection of proliferation, differentiation, and stress adaptation [113]. Whole-exome sequencing has uncovered that rare missense mutations in INO80D are predicted to compromise remodeler activity and are associated with a spectrum of vascular disorders encompassing aortic hypoplasia, premature atherosclerosis, and arterial stiffening [114]. These provide the first direct genetic evidence that impaired INO80-mediated chromatin accessibility predisposes to human vascular disease. Another study further showed that specific deletion of Ino80 in two major coronary progenitor tissues (venous sinus and endocardium) resulted in coronary vascular developmental defects [113]. Together, these findings establish INO80 as an epigenetic checkpoint linking chromatin plasticity to coronary patterning and underscore the potential therapeutic value of modulating INO80 remodeler activity in vascular developmental disorders.

### 5.2. Histone Modification in VSMC Phenotype Switching

#### 5.2.1. Histone Acetylation in VSMC Phenotype Switching

Within the histone acetyltransferase (HAT) family of proteins, p300 and CREB binding protein (CBP) are extensively studied histone acetyltransferases, frequently regarded as a single entity due to their substantial structural similarity [115]. However, a recent study has demonstrated that their expression patterns in carotid artery ligation-induced injured arteries diverge, exerting opposing regulatory effects on the VSMC phenotypic switching. In normal arteries, p300 facilitates the binding of H3K9Ac, H3K27Ac, and RNA polymerase II at the promoters of contractile genes. It also promotes interaction with ten-eleven translocation 2 (TET2), thereby establishing an open chromatin conformation conducive to the maintenance of vascular contractility. Conversely, although CBP exhibits histone acetyltransferase activity, it preferentially associates with genes related to synthesis and migration. In injured vessels, CBP expression is significantly upregulated, recruiting HDACs while counteracting TET2 methylation. This inhibits the expression of contractile genes and enhances the expression of synthetic genes by increasing H3K27Ac levels at these loci [8]. In addition, lysine acetyltransferase 2B (KAT2B), recruited by the staphylococcal nuclease domain-containing protein 1 (SNF) during wire-induced vascular injury, binds to the promoters of proliferation-related genes (Cdk2, Fos, and Cdk6) and migration-related genes (Rock1, Rock2, and Iqgap1) [70]. This binding promotes the acetylation of histone H3K27 and H3K9, thereby increasing chromatin accessibility and facilitating the phenotypic transition of smooth muscle cells to a synthetic phenotype.

HDACs are key enzymes that condense chromatin and repress gene transcription by removing acetyl groups from lysine residues on histones. Based on sequence homology and catalytic mechanism, HDACs are classified into classes I–IV [116]. The four HDAC classes exert distinct, often antagonistic, influences on VSMC phenotypes via targeted epigenetic events. Early studies have found that PDGF-BB-induced KLF4 recruits HDAC2, HDAC4, and HDAC5 to the CArG region of contractile genes in rat aortic SMCs, thereby inhibiting the transcriptional regulation of these genes by SRF and MYOCD [117]. Recent studies have progressively unveiled the role of HDACs in VSMC phenotypic switching, particularly focusing on HDAC3. Zhang et al. [118] elucidated that the long non-coding RNA ANRIL interacts with WD repeat-containing protein 5 (WDR5) and HDAC3 to form complexes that suppress the levels of H3K4me3 and H3K9ac at the promoter region of the NADPH oxidase 1 (NOX1) gene. This suppression consequently elevates reactive oxygen species (ROS) levels, facilitating the phenotypic transition of human aortic smooth muscle cells (HASMC) during the oxidized low-density lipoprotein (ox-LDL)-induced phenotypic transition of smooth muscle cells. A recent review summarized the role and mechanisms of HDAC3 in atherosclerosis [119]. Notably, HDAC1-3 exhibit dual enzymatic activities, functioning as both deacetylases and delactylases. Specifically, they catalyze the removal of lactyl groups from lysine residues, thereby mediating the delactylation of lactylated proteins [120]. The activation of HDAC3 significantly mitigates oncogenic Ras-induced smooth muscle senescence by inhibiting H4K12 lactate modification of the senescence-associated secretory phenotype (SASP) promoter [121]. Furthermore, HDACs are capable of catalyzing the acetylation modification of non-histone proteins as well. Specifically, in vitamin D3-induced vascular calcification, the influence of HDAC4 on the acetylation of Runt-related transcription factor 2 (RUNX2) within the cytoplasm is reduced, facilitating the translocation of RUNX2 to the nucleus. This translocation subsequently leads to the upregulation of OPN gene transcription, which in turn promotes the osteogenic transformation of rat aortic SMCs and accelerates vascular calcification [122]. In rat mesenteric arterial SMCs, increased HDAC4 by PDGF-BB stimulation activates the p38 mitogen-activated protein kinase (p38MAPK) and heat shock protein 27 (HSP27) signaling pathways, which regulate NOX activity and ROS production [123]. HDAC4 inhibition by a class IIa HDAC inhibitor, MC1568, alleviates neointimal formation in the ligated carotid artery of mice. The synergistic interplay among histone-modifying enzymes, chromatin-remodeling factors, and noncoding RNAs has also been explored. Research findings indicate that during the pathogenesis of thoracic aortic aneurysms, the HDAC9-MALAT1-BRG1 complex exerts a regulatory effect by increasing H3K27me3 levels, which in turn suppresses the chromatin accessibility of contractile genes [9]. Genetic ablation of HDAC9 or MALAT1 results in the reactivation of contractile gene expression in smooth muscle cells and mitigates the progression of aortic aneurysm formation.

Sirtuins (SIRTs) constitute a conserved family of NAD^+^-dependent protein deacetylases, classified as Class III HDACs [124]. Among them, SIRT1 has been extensively investigated in the context of the VSMC phenotypic switching [125]. In vascular injury induced by vascular ligation and guide wire injury, SIRT1 inhibits neointima formation and exerts a protective effect. SIRT1 deacetylates the AP-1 subunits c-Fos and c-Jun, preventing their binding to cyclin D1 and MMP-9 promoters and thereby silencing synthetic gene expression. Thus, SIRT1 functions to inhibit the proliferation and migration of VSMCs, while also preventing phenotypic shift towards a synthetic state in vascular mechanical injury [126]. Subsequently, in the investigation of the protective effects of energy restriction on abdominal aortic aneurysms (AAA), researchers observed that reduced SIRT1 deacetylase activity led to increased H3K9ac levels in the Mmp2 promoter, thereby exacerbating the progression of AAA [127]. In addition, decreased SIRT1 expression, which correlates with hypomethylation of the SIRT1 promoter, is also observed in HASMCs stimulated by calcium and phosphate. A reduction in H3K9 acetylation levels at the RUNX2 and osterix promoters by SIRT1 attenuated the osteogenic trans-differentiation of SMCs [128]. SIRT3 is the sole deacetylase enzyme found within mitochondria, where it plays a critical role in regulating mitochondrial metabolic homeostasis [129]. In angiotensin II (Ang II)-induced abdominal aortic aneurysm and neointima formation after carotid artery ligation in mice, the absence of SIRT3 results in the accumulation of mitochondrial acetyl-CoA, which, upon translocation to the nucleus, enhances H3K27 acetylation at the KLF4 promoter region. Finally, KLF4 Overexpression induces VSMC phenotypic switching, thereby mitigating vascular remodeling [130].

Histone acetylation is governed not only by acetyl-transferases and deacetylases, but also by the availability of the shared substrate, acetyl-CoA. In VSMCs, the canonical source of nuclear acetyl-CoA is the ATP-citrate lyase (ACLY)-mediated cleavage of cytosolic citrate. Pharmacologic or genetic blockade of ACLY dampens this supply and, consequently, blunts mechanically induced synthetic gene expression, underscoring the metabolic control of phenotype [131]. Citrate itself is actively trafficked across membranes. The progressive ankylosis protein ANK operates as a citrate exporter [132]. Wu et al. [133] found ANK was markedly down-regulated in AAA, resulting in cytosolic citrate overload. The abnormal citrate metabolism induces histone acetylation of inflammatory molecules CCL2, IL-6, and MMPs at H3K23, H3K27, and H4K5. This selective acetylation facilitates the phenotypic transition of smooth muscle cells towards an inflammatory state. Therefore, examining VSMC phenotypic switching through the framework of metabolic transport offers novel insights into the mechanism of VSMC phenotypic switching.

#### 5.2.2. Histone Methylation in VSMC Phenotype Switching

Histone methyltransferases and demethylases translate extracellular cues into durable chromatin states that dictate SMC identity. The SET and MYND domain-containing protein 2 (SMYD2) functions as a lysine methyltransferase that regulates both histone and non-histone methylation modifications. It plays a significant role in the pathological progression of tumors, inflammation, and cardiovascular diseases [134,135,136]. In neointima hyperplasia and vascular remodeling induced by carotid artery ligation, SMYD2 is reduced. In primary VSMCs from mice, SMYD2 is recruited by MYOCD to bind to the CArG region of the smooth muscle contractile gene promoters. This interaction promotes H3K4me3, resulting in an open chromatin structure near these genes, thereby inhibiting the phenotypic transition of smooth muscle cells towards a synthetic phenotype [137]. However, another study demonstrates a different expression and function of SMYD2 in vascular remodeling, upregulated SMYD2 in guidewire-injured carotid arteries and PDGF-BB-treated HASMCs leads to the induction of H3K36me3 and the promotion of HDAC3 expression [138]. HDAC3 subsequently binds to SRF, enhancing the expression of proliferation-related target genes, specifically cyclin-dependent kinase 4 (CDK4) and CDK6, which in turn promotes the synthetic phenotype of smooth muscle cells.

Enhancer of zeste homolog 2 (EZH2) is a key component of the core catalytic subunit of the polycomb repressive complex 2 (PRC2), which is instrumental in the repression of gene expression through the catalysis of mono-, di-, and trimethylation of H3K27 [139]. The involvement of EZH2 in smooth muscle phenotypes has been previously reviewed [140]. Recent investigations have demonstrated that both EZH2 and H3K27me3 are significantly upregulated in models of guidewire-induced carotid artery injury, leading to the inhibition of the cyclin-dependent kinase inhibitor p16^INK4A^ and the promotion of smooth muscle cell proliferation [141]. In atherosclerosis models, EZH2 targets senescence-associated genes such as P16 and P21, as well as anti-migration gene tissue inhibitors of metalloproteinases 2 (TIMP2) and TIMP3, which accelerate VSMC proliferation and migration within atherosclerotic plaques. Conversely, inhibitors of EZH2 have been shown to reduce the transition of smooth muscle cells to synthetic or osteogenic phenotypes [142,143]. Protein arginine methyltransferase 5 (PRMT5) is the major type-II PRMT that catalyzes symmetric dimethylation of arginine residues (SDMA) on histones and non-histone substrates [144]. Zhu et al. [145] demonstrated that PRMT5 catalyzes the di-methylation of histones H3R8 and H4R3, which subsequently inhibits the acetylation of H3K9 and H4 on SMA and SM22 promoters in HASMC. This inhibition ultimately restricts the binding of the SRF/MYOCD/p300 complex to the CArG boxes of smooth muscle cell marker genes. Furthermore, the smooth muscle-specific knockout or pharmacological inhibition of PRMT5 with EPZ015666 attenuates neointimal formation induced by carotid artery ligation in mice and carotid balloon injury in rats. Additionally, PRMT5 also participates in the inflammatory transformation of smooth muscle cells. In primary rat and human VSMCs with trimethylamine N-oxide (TMAO) treatment, PRMT5 facilitates the methylation of NF-κB p65 and promotes the expression of vascular cell adhesion molecule 1 (VCAM-1), therefore promoting the inflammatory phenotypic transformation of VSMC [146].

It has been reported that H3K9me2/3 is associated with the inflammatory response in vascular smooth muscle cells and is implicated in metabolic memory [147,148]. G9a, a member of the SET family of histone methyltransferases, is responsible for catalyzing H3K9 monomethylation and dimethylation [149]. It plays a crucial role in embryonic development and tissue differentiation by regulating epigenetic processes. Upon interferon gamma exposure, G9a collaborates with HDAC2 to form repressive complexes that suppress collagen type I alpha 2 chain (COL1A2) expression in smooth muscle cells, contributing to atherosclerotic plaque destabilization [150]. Furthermore, G9a promotes the phenotypic transition of smooth muscle cells towards a synthetic phenotype in response to mechanical injury. G9a targets the promoter region of smooth muscle contractile genes, where it catalyzes H3K9me2 in the presence of the transcription factor BTB domain and CNC homolog 1 (BACH1), thereby maintaining a repressed chromatin state of smooth muscle contractile genes [75]. The suppressor of variegation 3-9 homolog 1 (SUV39H1) is an enzyme involved in histone methylation and heterochromatin formation, specifically catalyzing the trimethylation of H3K9me3 [151]. Prior research has demonstrated that SUV39H1 exerts anti-inflammatory protective effects in the vasculature of diabetic murine models [152]. In a carotid artery injury model, SUV39H1 has been identified as a pivotal factor in facilitating the phenotypic switching of smooth muscle cells towards a synthetic state [153]. In human coronary artery smooth muscle cells upon PDGF-BB stimulation, SUV39H1 expression is upregulated, leading to an increase in H3K9me3 levels at promoter regions of genes associated with smooth muscle cell contraction. Conversely, enhanced deposition of SUV39H1 at the KLF4 promoter results in the suppression of DNA methyltransferases 1 (DNMT1) and 5-methylcytosine (5mC) levels, promoting KLF4 transcription and subsequent cell dedifferentiation. This elucidates a critical mechanism by which SUV39H1 mediates smooth muscle cell phenotypic transformation through the dynamic modulation of H3K9me3 [154]. Additionally, another study has suggested an alternative regulatory pathway wherein SUV39H1 influences neointimal formation by modulating the expression of the transcription factor hypermethylated in cancer 1 (HIC1), which in turn affects the transcription of jagged1 [155]. The application of F5446, a specific small molecule inhibitor of SUV39H1, significantly improved neointima formation after vascular injury, further broadening the ability of SUV39H1 as a potential target.

Compared with methyltransferases, the role of demethylases in VSMC phenotypic switching has been far less explored. Jumonji domain-containing 2A (JMJD2A/KDM4A), a JmjC-domain lysine-specific demethylase, removes the repressive H3K9me3 and the elongation-associated H3K36me3 marks [156]. Hu et al. [157] found that JMJD2A, which is upregulated in RASMCs stimulated by AngII, catalyzes demethylation of H3K9me3 in the promoter of cell cycle-related proteins (cyclin D1 and p21). This enzymatic activity promotes the proliferation of smooth muscle cells and drives their transition to a synthetic phenotype. A pharmacologic blockade with the selective inhibitor IOX1 restores H3K9me3, confirming the enzyme’s causal role in the Ang II response. Likewise, KDM2B is markedly induced in inflammatory VSMCs and human AAA specimens. By docking at the promoters of contractile genes, KDM2B demethylates H3K4me3 and H3K36me2, thereby silencing these lineage markers and accelerating the shift toward a synthetic, pro-inflammatory phenotype [158] (Table 1).

### 5.3. DNA Methylation in VSMC Phenotype Switching

DNA methylation progressively shifts toward a hypermethylated state as atherosclerosis advances [167]. In mammals, DNA methylation is principally orchestrated by two families of enzymes: the de novo DNMT3a and DNMT3b, and the maintenance enzyme DNMT1 [168]. DNMT1, phosphorylated and stabilized by G protein-coupled receptor kinase 2 (GRK2) to evade ubiquitin-proteasomal degradation, accumulates in VSMCs following exposure to guidewire trauma or PDGF-BB [169]. This accumulation guides DNMT1 towards the promoters of contractile genes, leading to their silence and the acceleration of the synthetic phenotype. Simultaneously, DNMT1 methylates the TET2 promoter, disrupting the methylation and demethylation equilibrium. Pharmacologic blockade with the DNMT inhibitor 5-aza-2′-deoxycytidine (5-Aza) lifts TET2 repression, boosts 5-hmC occupancy at the myocardin promoter, restores myocardin expression, and re-establishes the contractile phenotype [170]. Additionally, Zhong et al. [171] found that DNMT1 and TET2 jointly regulate miR-145. The latter targets NOD-like receptor family pyrin domain containing 3 (NLRP3), thereby inhibiting the VSMC inflammatory response. H19 induces aortic valve calcification and the osteogenic differentiation of valve interstitial cells by inhibiting Notch1 signaling and increasing Runx2 expression. In the context of vascular calcification, the transcriptional activity of H19 is inhibited by DNA methylation mediated by DNMT3b [165]. This regulatory mechanism leads to the inhibition of RUNX2-induced osteogenic differentiation of smooth muscle cells.

**Figure 3:**
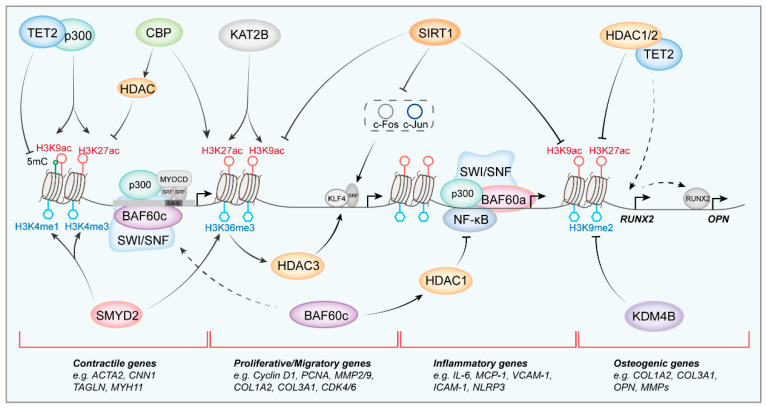
Chromatin-remodeling code that gates VSMC phenotypic genes. Expression of contractile marker genes is co-regulated by multiple histone-modifying enzymes and the MYOCD-SRF complex. p300 associates with H3K9Ac and H3K27Ac at contractile promoters and interacts with TET2 to maintain an open chromatin state. In contrast, CBP recruits HDACs to inhibit contractile gene expression. Furthermore, CBP promotes H3K27Ac near synthetic and migration genes, thereby driving the synthetic phenotype [8]. H3K27 and H3K9 acetylation at KAT2B-bound pro-proliferation/migration loci also drives VSMC synthetic switching [70]. MYOCD recruits SMYD2 to CArG elements of contractile promoters, where SMYD2-catalyzed H3K4me3 establishes a transcriptionally permissive chromatin state [137]. Following vascular injury, SMYD2 is transcriptionally up-regulated and deposits H3K36me3, thereby inducing HDAC3 expression. HDAC3 physically interacts with SRF to activate proliferative genes [138]. BAF60a strengthens BRG1 occupancy on NF-κB loci, driving SMC inflammation [111]. BAF60c normally sustains the contractile state via SRF-p300 assembly and maintains NF-κB loci in a HDAC1-repressed configuration [112]. SIRT1 governs VSMC plasticity by deacetylating AP-1 (c-Fos/c-Jun), thereby blocking its occupancy of proliferative gene promoters [126]. Moreover, SIRT1 inhibits H3K9ac at osteogenic gene RUNX2 and a series of synthetic genes and restrains trans-differentiation of SMC [128]. RUNX2 transcription is simultaneously restrained by KDM4B-mediated H3K9me2 demethylation [179] and the dual repressive actions of HDACs and TET2 enzymes [166].

The histone demethylase TET2 catalyzes the oxidation of methylcytosine to generate five hydroxymethylcytosines (5hmC), thereby facilitating gene activation and playing a crucial role in the VSMC differentiation [172]. TET2 activity is upregulated by rapamycin induction and downregulated by PDGF intervention. TET2 associates with the promoters of key transcription factors, SRF and MYOCD, and sustains the VSMC contractile phenotype by increasing 5hmC levels in the binding regions and regulating chromatin accessibility [173]. Notably, TET2 expression was significantly reduced in a femoral artery wire injury model, while local injection of a TET2-overexpressing virus into the injured tissue markedly improved intimal hyperplasia [173]. In facilitating the crosstalk of various cells, endothelium-derived TET2 mRNA and protein are transported to adjacent medial cells, where they suppress KLF4 expression and phenotype switching from the contractile state to the synthetic phenotype [174]. In addition, TET2 also frequently assembles into complexes with other key regulatory factors to jointly modulate gene expression [175]. He et al. [166] investigated the formation of an inhibitory regulatory complex involving TET2, HDAC1/2, and SMAD nuclear interacting protein 1 (SINP1) in HASMC and vascular calcification. TET2 interacts with HDAC1/2, facilitating their recruitment to the RUNX2 promoter, which subsequently inhibits RUNX2 transcription by deacetylating H3K27ac, thereby preventing the transition of smooth muscle cells to an osteogenic phenotype. Conversely, significant downregulation of TET2 in calcified tissues led to the overexpression of RUNX2, exacerbating vascular calcification. Apart from its impact on the synthetic and osteogenic phenotype of SMCs, TET2 is intricately associated with vascular inflammation. Exome sequencing has revealed that carriers of Tet gene mutations exhibit elevated levels of overall inflammation [176]. In TET2 mutant-clonal hematopoiesis of indeterminate potential (CHIP) mice, increased levels of IL-1β and NLRP3 in monocyte-macrophages contribute to accelerated plaque formation [177]. Bone marrow transplantation experiments have demonstrated that the deletion of hematopoietic TET2 is sufficient to induce arterial lesions. The reduction in the levels of the NLRP3 inflammasome pathway, along with cleaved Caspase-1 and IL-1β protein expression in VSMCs, has been observed with TET2 overexpression in RASMC [178]. Consequently, the DNA methylation network links metabolic or mechanical signals to DNA methylation dynamics, determining VSMC destiny and participating in the progression of vascular diseases (Figure 3).

### 5.4. High-Order Chromatin Organization in VSMC Phenotype Switching

With the rapid advancement of bioinformatics technologies, the role of high-order chromatin organization in diseases is being progressively deciphered [180]. The current research landscape remains largely focused on developmental and differentiation systems, as well as cancers and diseases associated with genetic mutations [181,182,183]. Notably, a recent study has elucidated the crucial role of alterations in three-dimensional chromatin architecture within VSMCs. LEM domain-containing protein 3 (LEMD3) is an inner nuclear membrane protein that is anchored to the inner nuclear membrane via its LEM domain and interacts with the nuclear lamina [184]. The study by Li et al. identified LEMD3 as a potential regulator of VSMC phenotypic switching through a CRISPR-based genetic knockout screening strategy [185]. In tamoxifen-induced smooth muscle-specific LEMD3 knockout mice, the expression of smooth muscle contractile marker genes ACTA2, CNN1, and TAGLN was significantly reduced in the aorta. Additionally, this genetic ablation exacerbated phenotypic switching and neointimal hyperplasia in the carotid wire injury model. At the mechanistic level, the researchers demonstrated that LEMD3 interacts with chromobox protein homolog 3 (CBX3), a principal heterochromatin recognition protein, to anchor heterochromatin at the nuclear periphery, thereby preserving the three-dimensional chromatin architecture. This structural maintenance is crucial for the contractile phenotype of VSMCs. Through high-throughput/resolution chromosome conformation capture (Hi-C) and whole-chromosome simulations and the 3D whole-nucleus maximum entropy model, the study found that LEMD3 deficiency leads to the repositioning of heterochromatin from the nuclear periphery to the nuclear interior and enhances interactions between TADs at the boundaries of A and B compartments, resulting in downregulation of contractile-related gene expression and ultimately loss of the contractile phenotype. Notably, the results of the integrative analysis revealed a negative correlation between the signals identified by ATAC-Seq and the interactions between TADs. This finding underscores the association between alterations in high-order chromatin conformation and the regulation of chromatin accessibility and gene expression in VSMCs, thereby offering a novel paradigm for elucidating the mechanisms underlying VSMC phenotypic transition.

## 6. Targeting Chromatin Remodelers

Chromatin-remodeling enzymes have risen to prominence as key epigenetic modulators of VSMC phenotypes. The inherent reversibility of epigenetic modifications endows these enzymes with considerable therapeutic potential. A wide array of epigenetic agents is currently under investigation in clinical trials, with several—such as the pan-HDAC inhibitors vorinostat and panobinostat—already approved by the FDA for treating hematologic malignancies [186]. Within the realm of vascular remodeling-associated diseases, potential chromatin remodeling molecular targets, as previously described, have been identified through investigations in animal models and cellular systems. These potential targets are summarized in Table 2.

### 6.1. Epigenetic Drugs

Broad-spectrum dietary HDAC inhibitors were the first to demonstrate potential. Butyrate resets site-specific post-translational modifications on histone H3 and establishes a self-regulating acetylation code. It inhibits VSMC proliferation by regulating G1-specific cell cycle proteins [212]. Valproic acid, another pan-HDAC inhibitor, displaces RNA polymerase II and the acetyltransferase p300 from the Nox2/4/5 promoters, resulting in the removal of H3K4me3 and H3K9ac, which alleviates pulmonary hypertension [213]. Next, more selective agents now facilitate isoform-specific interrogation. The endogenous class I inhibitor β-hydroxybutyrate inhibits HDAC1, preserves contractile markers, and sustains the differentiated phenotype [214]. Synthetic inhibitors of class I (MS-275) and class IIa (MC-1568) mitigate elastin degradation and slow the progression of experimental AAA by reducing IL-6, MMP-2, and MMP-9 levels [215]. Another class IIa inhibitors TMP269 and TMP195 suppress HDAC5, diminish angiotensin-II-induced ROS production, and alleviate maladaptive remodeling [192]. By inhibiting HDAC3, RGFP966 inhibited PA-induced lipid droplet deposition, proliferation and migration of SMCs [191]. Zhong et al. showed that the combined inhibition of the methyltransferase SMYD2 using LLY-507 and HDAC3 using RGFP966 disrupts the SMYD2-HDAC3-SRF signaling axis [138]. This disruption effectively limits neointimal hyperplasia following vascular injury. In addition, the class III histone deacetylase SIRT1 is pharmacologically controlled by the activator resveratrol and the inhibitor EX-527 [216,217]. Its selective agonist SRT2104 up-regulates TSC2, silencing mTORC1, curbing extracellular-matrix synthesis, suppressing proliferation, and triggering apoptosis [198]. Collectively, these effects contribute to the improvement of vascular remodeling.

Different from the protective effect, the DNMT1 inhibitor 5-Aza significantly enhances the osteogenic differentiation of smooth muscle cells. This is evidenced by an increase in osteoblastic markers such as MMP3, bone morphogenetic protein (BMP2), and RUNX2, which subsequently leads to reduced vascular elasticity and contributes to vascular stiffness [218].

Beyond enzymes that “write” or “erase” histone marks, the epigenetic “reader” bromodomain-containing protein 4 (BRD4) deciphers acetylation signatures and orchestrates transcription and co-transcriptional splicing by scaffolding acetylated histones, transcription factors and splicing regulators [219]. BRD4 is significantly elevated during the process of neointima formation. The selective inhibition of BRD4 using JQ-1 effectively reduces intimal hyperplasia, primarily by inhibiting the VSMCs’ proliferation and migration [220].

Despite the promising therapeutic effects demonstrated by numerous small-molecule drugs in preclinical studies, their lack of target specificity often leads to a range of adverse reactions, which significantly restricts their clinical application. Therefore, the field of epigenetic therapies requires further investigation and development.

### 6.2. Genetic-Editing Tools

Unlike conventional pharmaceuticals, gene editing technology enables precise targeting of specific genomic loci, thereby minimizing off-target effects associated with traditional drugs and enhancing the specificity of therapeutic interventions [221]. The clustered regularly interspaced short palindromic repeats (CRISPR)/Cas9 system is the most widely adopted genome-editing tool. It is composed of the DNA-cleaving Cas9 nuclease and a single-guide RNA (sgRNA) that specifies the genomic target. This system relies on endogenous DNA-repair pathways (non-homologous end-joining or homology-directed repair), events that frequently generate insertions or deletions. Substitution of catalytically inactive Cas9 (dCas9) for wild-type Cas9 abolishes cleavage while preserving programmable DNA binding, thereby furnishing a basis for new CRISPR applications.

Coupling dCas9 to histone-modifying or DNA-methylating enzymes yields CRISPR-ON/OFF switches that regulate gene expression through epigenetic marks, which is an approach to avoid permanent DNA sequence changes and enhance the safety profile of gene-therapy strategies [222,223]. The fusion of dCas9 with p300 increases histone acetylation levels at specific genomic sites, thereby fully activating enhancers and promoters and consequently upregulating gene expression [224]. In addition, fusing dCas9 with DNMT3A, DNMT3B, or TET enables these DNA methylation-modifying enzymes to be precisely targeted to specific DNA methylation sites, thereby modulating chromatin conformation and gene expression [225,226].

Delivery remains a bottleneck for vascular applications because the endothelial barrier limits access of CRISPR–Cas9 components to the vessel wall. Zhang et al. first overcame this hurdle by combining cholesterol-terminated ethanolamine-aminated poly (glycidyl methacrylate) nanoparticles with angiotensin-II-assisted intervention to achieve Fbn1 disruption in the aortic endothelium of adult mice [227]. Subsequently, another group employed poly (ethylene glycol)-block-poly (lactic-co-glycolic acid) nanoparticles to co-deliver Cas9 mRNA and a plasmid harboring a Cadherin 5 (CDH5) promoter-driven expression frame plus U6-driven sgRNA [228]. Efficient targeted genome editing of vascular endothelial cells in multiple organs of adult mice has been achieved.

For functional studies, adeno-associated virus (AAV) vectors are commonly harnessed to supply CRISPR/Cas9 machinery [229]. AAV-mediated somatic editing of low-density lipoprotein receptor (Ldlr) in Ldlr-mutant mice markedly attenuated atherosclerosis, offering a therapeutic avenue for familial hypercholesterolemia [230]. More recently, Mao and colleagues used lipid nanoparticles (LNPs) to deliver mRNA encoding an epigenetic editor to the liver. Incorporation of human DNMT3L and the ZIM3 KRAB domain significantly enhanced proprotein convertase subtilisin/kexin type 9 (PCSK9) silencing efficiency. Concurrent deposition of DNA methylation and H3K27me3 at the target promoter produced durable, allele-specific repression [231]. These preliminary preclinical studies demonstrate the specificity and high efficiency of gene-editing and drug delivery technologies in blood vessels and their effectiveness in vascular diseases, showing the potential application of epigenetic remodeling molecules. However, further in-depth exploration is still needed to evaluate their efficacy and safety in humans.

## 7. Conclusions and Future Perspectives

Chromatin remodeling transduces hemodynamic, metabolic, and inflammatory signals into decisive epigenetic cues that drive the multidirectional phenotypic switching of smooth muscle cells and thereby governs vascular remodeling. The ATP-dependent remodeling complex determines whether key transcription factors such as SRF, MYOCD, RUNX2, and KLF4 can access CArG or TGF-β response elements by transient sliding or popping nucleosomes, thus rapidly initiating the phenotypic conversion process. Next, histone modification and DNA methylation enzymes provide reversible “chemical tags” that write mechanical stress, oxidative stress, or calcium and phosphorus microenvironment signals into chromatin states and form phenotypic memories.

At present, pharmacological or genetic interventions targeting chromatin-remodeling molecules have been validated in pre-clinical models to effectively attenuate vascular remodeling processes such as vascular calcification and neointimal hyperplasia. Nevertheless, their clinical translation is constrained by unresolved challenges concerning delivery efficiency, safety, and tissue specificity. Among them, HDAC inhibitors exhibit greater translational potential given their established approval for oncological indications and the most extensive preclinical evidence base in cardiovascular disease. In addition, CRISPR-based epigenome editing emerges as the most strategically compelling therapeutic avenue, attributable to its capacity for locus-specific chromatin modification and targeting specificity. Recent attempts to achieve localized delivery of therapeutic proteins to injured arterial segments, such as adventitial stent coatings [232], remain confined to animal experimentation and constitute a pivotal research frontier for the forthcoming years. Moreover, the complexity and multidirectionality of SMC phenotype switching imply that the lineage origin and the interactive dynamics among SMC subpopulations undergoing divergent differentiation trajectories are still obscure. The continued evolution of lineage-tracing strategies, spatial transcriptomics, and single-cell multi-omics technologies, however, promises to trace the developmental path of SMCs and analyze their gene expression patterns at different developmental stages. This integrated analysis helps to reveal the heterogeneity of SMCs and their dynamic changes under different pathological conditions, and comprehensively reveals the spatiotemporal dynamics of smooth muscle cell differentiation. Combined with gene editing technology, we can precisely modify specific genes in SMC to study their role in diseases.

## Figures and Tables

**Table 1:** Histone modifications in VSMC phenotypic switching.

Proteins	Study Model	Species	Expression	Types of Histone Modifications	Mechanism	Reference
Methylation						
SMYD2	carotid artery ligation model	mice	downregulated	H3K4me3	Promoted contractile gene level	[137]
	carotid artery wire injury model	mice	upregulated	H3K36me3	Promoted HDAC3 expression and formed the HDAC3-SRF axis	[138]
SUV39H1	carotid artery ligation and femoral artery wire injury	mice	upregulated	H3K9me3	Regulated HIC1-Jag1 axis	[155]
	carotid artery ligation model	mice	upregulated	H3K9me3	Inhibited contractile gene expression and induced KLF4	[154]
	carotid artery balloon injury model	rat	upregulated	H3K9me3	Inhibited p21 and p27Kip1 and promoted ID3 level	[153]
	db/db	mice	downregulated	H3K9me3	Inhibited inflammatory genes	[152]
	STZ-induced DM/carotid artery balloon injury model	rat	downregulated	H3K9me3	Promoted phosphor-ERK1/2 and complement C3	[159]
EZH2	Carotid artery wire injury model	rat	upregulated	H3K27me3	Inhibited the transcription of p16^ink4a^	[141]
	HFD-fed ApoE^−/−^ model	mice	upregulated	H3K27me3	Reduced the transcription of P16, P21, and TIMP3	[143]
	BAPN-induced TAD model	mice	upregulated	/	Suppressed Itgb3 expression	[160]
G9a	HFD-fed ApoE^−/−^ and carotid artery ligation model	mice	downregulated	H3K9me2	Inhibited MMP3, MMP9, MMP12, and IL6	[147]
DOT1L	HFD-fed ApoE^−/−^ model	mice	upregulated	H3K79me2	Promoted NF-κB1 and NF-κB2 transcription	[161]
PRMT5	carotid artery ligation model and carotid balloon injury model	mice/rat	upregulated	H3R8me2 and H4R3me2	Suppressed contractile genes	[145]
	carotid artery wire injury model	mice	upregulated	/	Demethylated p65 on arginine 30	[146]
KDM1A	carotid artery wire injury model	mice	upregulated	H3K4me2	Inhibited p21 expression	[162]
	aortic endothelial balloon injury	rat	upregulated	/	Inhibited BMP-2 expression	[157,163]
KDM2B	PPE-induced AAA model	mice	upregulated	H3K4me3 and H3K36me2	Suppressed contractile genes transcription	[158]
KDM4A	AngII-stimulated VSMC	/	upregulated	H3K9me3	Increased cyclin D1 expression and inhibited p21 expression	[126]
	carotid artery ligation model	mice	downregulated	H3K9me3	Promoted contractile genes expression	[154]
KDM6B	carotid artery wire injury model and carotid artery balloon injury model	mice/rat	upregulated	H3K27me3	Promoted NADPH oxidase 4 expression	[164]
Acetylation						
CBP	carotid artery ligation and femoral artery wire injury	mice	upregulated	H3K27ac	Recruited HDACs and inhibited contractile genes	[8]
p300	carotid artery ligation and femoral artery wire injury	mice	downregulated	H3K27ac and H3K9ac	Promoted contractile genes and inhibited synthetic genes	[8]
KAT2B	carotid artery wire injury model and femoral artery wire injury model	rat/mice	upregulated	H3K27ac and H3K9ac	Promoted Cdk2, Fos, and Cdk6, Rock1, Rock2, and Iqgap1	[70]
SIRT1	carotid artery ligation or carotid artery wire injury model	mice	downregulated	/	Decreased the induction of cyclin D1 and MMP9	[126]
SIRT1	AngII-infused ApoE^−/−^ mice	mice	downregulated	H3K9ac	Inhibited MMP2	[127]
SIRT1	HFD-fed ApoE^−/−^ model	mice	downregulated	H3	Inhibited RUNX2	[165]
SIRT1	/	/	downregulated	H3K9ac	Reduced RUNX2	[128]
SIRT3	Ang II-infused ApoE^−/−^ mice and carotid artery ligation model	mice	downregulated	H3K27ac	Suppressed KLF4	[130]
HDAC1/2	vitamin D3 and adenine diet-induced CKD model	mice	downregulated	H3K27ac	Suppressed RUNX2	[166]
HDAC3	HFD-fed ApoE^−/−^ model	mice	downregulated	H4K12la	Inhibited SASP transcription	[121]

**Table 2:** Small-molecule inhibitors targeting epigenetic remodeling enzymes in vascular diseases.

Drugs	Types	Function	Disease/Pathology	References
Trichostatin A (TSA)	Pan-HDAC inhibitor	Increased expression of TAL1 target genes and improved the vascular repair function	Vascular restenosis	[187]
		Inhibited osteogenic transition of VSMCs and vascular calcification	Vascular calcification	[188]
Butyrate	Pan-HDAC inhibitor	Inhibited osteogenic transition of VSMCs and vascular calcification	Vascular calcification	[188]
Entinostat	HDAC1 inhibitor	Repressed CTH transcription and alleviated AAD	Aneurysm and aortic dissection	[189]
MS-275	HDAC1-3 inhibitor	Enhanced endothelial and vascular permeability	Vascular inflammation	[190]
RGFP966	HDAC3 inhibitor	Reversed PA-induced VSMC phenotypic transition	-	[191]
		Reduced VSMC proliferation and neointima formation after injury	Vascular restenosis	[138]
TMP269/TMP195	HDAC5 inhibitor	Inhibited ROS generation and vascular hypertrophy	Hypertension	[192]
Tubastatin A	HDAC6 inhibitor	Increased SRF transcription and contractile protein expression	Vascular restenosis	[193]
		Suppressed cigarette smoke-stimulated bronchial and pulmonary arterial remodeling	Chronic obstructive pulmonary disease	[194]
LMK235	HDAC6 inhibitor	Inhibited vascular contraction and hyperplasia	Hypertension	[195]
PCI34051	HDAC8 inhibitor	Suppressed inflammation and retained vasoconstriction	Hypertension	[196]
TMP195	HDAC9 inhibitor	Mitigated the progression of established lesions and inhibited the infiltration of inflammatory cells	Atherosclerosis	[197]
SRT2104	SIRT1 activator	Inhibited ECM production, cell proliferation and apoptosis	Pulmonary hypertension	[198]
EX-527	SIRT1 inhibitor	Promoted apoptosis	Pulmonary hypertension	[199]
AGK2	SIRT2 inhibitor	Exacerbated PA-HG-induced pyroptosis	Diabetic angiopathy	[200]
GSK126	EZH2 inhibitor	Inhibited VSMC proliferation and migration	Hypertension	[142]
GSK343	EZH2 inhibitor	Activated autophagy and inhibited vascular calcification	Vascular calcification	[201]
		Restored contractile protein expression	Thoracic aortic aneurysms	[202]
LLY-507	SMYD2 inhibitor	Reduced VSMC proliferation and neointima formation after injury	Vascular restenosis	[138]
EPZ031686	SMYD3 inhibitor	Inhibited p21 level and alleviated vascular senescence	Vascular aging	[203]
5-Azacytidine	DNMT1 inhibitor	Reduced plaque area and increased SMC contractile gene expression	Vascular restenosis	[204]
5-Aza 2′-deoxycytidine	DNA methyltransferase inhibitor	Activated FoxM1 and improved endothelial regeneration and vascular repair in aged lungs	Acute respiratory distress syndrome	[205]
RG108	DNMT inhibitor	Increased arterial blood pressure	Hypertension	[206]
S-adenosylhomocysteine	DNA methyltransferase inhibitor	Protected against vascular senescence	Atherosclerosis	[207]
C35	TET inhibitor	Reduced arterial blood pressure	Hypertension	[206]
Pemetrexed	METTL4 antagonist	Alleviated repaired mitochondrial function and atherosclerotic progression	Atherosclerosis	[208]
JQ1	BET inhibitor	Prevented neointima formation and increased aortic stiffness	Vascular aging	[209]
		Inhibited the proliferation and migration of VSMCs	Vascular restenosis	[210]
		Suppressed TNFα-induced inflammation	Pulmonary arterial hypertension	[211]
ARV-825	BET inhibitor	Prevented neointima formation and increased aortic stiffness	Vascular aging	[209]

## Data Availability

Not applicable.

## Abbreviations

AAA	abdominal aortic aneurysms
AAD	aneurysm and aortic dissection
ACLY	ATP-citrate lyase
ACTA2	α-smooth muscle actin 2
ATF	activating transcription factor
CCL2	C-C motif chemokine ligand 2
CDK	cyclin-dependent kinase
CHD	Chromodomain Helicase DNA binding protein
ERK	extracellular signal-regulated kinase
EZH2	Enhancer of zeste homolog 2
HDAC	histone deacetylase
HSP27	heat shock protein 27
H3K27ac	H3 lysine 27 acetylation
IL-6	interleukin 6
KAT2B	lysine acetyltransferase 2B
KLF4	Krüppel-like factor 4
MMP	matrix metalloproteinase
MRTFA	myocardin-related transcription factor-A
MYH11	myosin heavy chain 11
NuRD	nucleosome remodeling and deacetylase
OPN	Osteopontin
ox-LDL	oxidized low-density lipoprotein
PDGF	platelet-derived growth factor
PLGA	poly(lactic-co-glycolic acid)
PRC2	polycomb repressive complex 2
PTM	post-translational modifications
RUNX2	Runt-related transcription factor 2
SEM-like	Stem/Endothelial/Monocyte-like
SMYD2	SET and MYND domain-containing protein 2
SRF	Serum-response factor
SUMO	small ubiquitin-related modifier
SUV39H1	suppressor of variegation 3-9 homolog 1
SWI/SNF	switch defective/sucrose non-fermentable
TET2	ten-eleven translocation 2
TGF-β	transforming growth factor-beta
TIMP	tissue inhibitors of metalloproteinases
VSMC	vascular smooth muscle cells
WDR5	WD repeat-containing protein 5
